# Editorial: Redox biology and crop health

**DOI:** 10.3389/fpls.2025.1583059

**Published:** 2025-03-24

**Authors:** Soumen Bhattacharjee

**Affiliations:** Department of Botany, Plant Physiology & Biochemistry Research Laboratory, University Grants Commission (UGC) Centre For Advanced Study, University of Burdwan, Burdwan, West Bengal, India

**Keywords:** plant redox biology, reactive oxygen species, crop health, defense mechanism, redox signaling, gene expression

A major characteristic of environmental stressors is their detrimental effect on redox biology through the disruption of redox homeostasis in crop plants (Mittler et al., 2022; Bhattacharjee, 2019). Crops respond to environmental changes for acclimation by utilizing a complex redox system. ROS generation, detox scavenging, sensing, oxidative deterioration, signaling and transcriptional reprogramming are all explained by redox biology. Crop plants have an elaborate antioxidative defense mechanism that not only contends with elevated ROS levels but also fine-tunes the endogenous redox cue to regulate different physiological and developmental processes (Riaz et al., 2022; Roy and Bhattacharjee, 2022). It is believed that the ROS wave resulting from the perception of unfavorable environmental cues combines with other metabolic and signaling pathways to allow for quick systemic acclimation and regulate crop health. Furthermore, metabolism, development, differentiation, stress signaling, interactions with other growth factors, and systemic responses in crops are all regulated by internal redox cues (Noctor et al., 2018; Bhattacharjee, 2012). Thus, Redox Biology occupies a central position in plant defense responses that regulate crop health (Mittler and Jones, 2024). Our understanding of defense signaling, which explores redox cues for stress acclimation and improvement of crop health has significantly improved in recent years (Mittler et al., 2022; Bhattacharjee, 2019). Therefore, it is expected that the mounting information on different domains of redox biology will further inspire efforts that leverage this information to improve crop health and food security (Cejudo et al., 2021; Kerchev et al., 2015).

The Research Topic, ‘*Redox Biology and Crop Health’* includes four research articles from scientists working in the field of crop redox biology, and provides new perspectives on how redox biology influences crop health. The importance of regulation of redox homeostasis through antioxidant buffering is crucial for the survival and performance of crops. In a research article, Wang et al. suggested that the decrease in antioxidant competence and glutathione biosynthesis triggered considerable ROS accumulation, leading to programmed cell death, which ultimately reduced yield components in a barley lesion mimic mutant (LMM 5386). Furthermore comparative transcriptomic investigation (RNA-seq) analysis identified genes that were differentially expressed in LMM 5386 compared to the wild type. The necessity of antioxidant-coupled redox buffering for crop performance and survival was strongly supported by GO and KEGG functional annotations of the transcriptome data, which indicated that lesion mimic formation in this barley mutant was mediated by pathways involving glutathione metabolism.

The effect of waterlogging on oxidative stress and associated damage, which strongly impact crop health was investigated by Umicevic et al. using two contrasting tomato genotypes [Trebinjski sitni (GB1126) and Žuti (GB1129)], which differed in their waterlogging sensitivity. This study provided strong evidence for the redox contribution to crop health, with a positive correlation found between waterlogging tolerance and antioxidant competence (peroxidase activity and phenolic compound content). Furthermore, this study revealed that waterlogging priming can induce stress memory by adjusting the content of bioactive secondary metabolic imprint phenolics in tissues, which are important for preserving redox homeostasis and, consequently, for reducing ROS damage in tomatoes when waterlogging recurs.

In their study, Li et al. examined the effect of chitosan on the cold stress response of *Kobresia pygmaea*, addressing the function of antioxidant systems, such as enzymes and non-enzymatic redox agentes, in addition to identifying genes linked to cold tolerance. The physiological response of *K. pygmaea* in terms of the accumulation of osmoregulatory compounds, photosynthetic features, and chloroplast architecture under the influence of chilling-induced altered redox cues and key gene expression was studied. The application of exogenous chitosan was found to enhance cold tolerance by modulating and restoring redox homeostasis while upregulating the activities of antioxidant defense systems and increasing the pool of soluble antioxidants, such as glutathione and ascorbate, which help the plant maintain the Rubisco activity and basal photosynthesis to optimize the carbohydrate metabolism. These findings provide essential theoretical points of reference for future studies on the use of chitosan for crops’ resistance to cold temperatures including plants of alpine origin.

In their article, Welle et al. examined NO emissions from the root systems of crops (tomato, tobacco, and barley) in response to low oxygen levels in the humidified medium of a root reactor. In addition, the contribution of NO derived from plants to the global NO budget during low-oxygen periods was calculated. The *in vivo* NO emissions of three experimental plant species under low oxygen conditions were analyzed under anoxic conditions and were found to be higher than those under hypoxic conditions. According to these authors, plants experiencing low-oxygen stress may be responsible for 1 to 9% of annual NO emissions, with implications for crop health.

In summary, the four studies included in this Research Topic contribute significantly to a better understanding of the redox regulatory mechanisms underlying plant stress responses and may suggest interesting new redox-based strategies for monitoring crop health and engineering crops with improved stress resistance.

## References

[B1] Bhattacharjee S. (2012). The language of reactive oxygen species signaling in plants. J. Bot. 2012, 985298. doi: 10.1155/2012/985298

[B2] Bhattacharjee S. (2019). “ROS in aging and senescence,” in Reactive oxygen species in plant biology, (Springer Nature India Private Limited) 65–79.

[B3] Cejudo F. J. González M. C. Pérez-Ruiz J. M. (2021). Redox regulation of chloroplast metabolism. Plant Physiol. 186, 9–21. doi: 10.1093/plphys/kiaa062 33793865 PMC8154093

[B4] Kerchev P. De Smet B. Waszczak C. Messens J. Van Breusegem F. (2015). Redox strategies for crop improvement. Antioxid. Redox Signal. 23, 1186–1205. doi: 10.1089/ars.2014.6033 26062101

[B5] Mittler R. Jones D. P. (2024). The redox code of plants. Plant Cell Environ. 47, 2821–2829. doi: 10.1111/pce.14787 38088476

[B6] Mittler R. Zandalinas S. I. Fichman Y. Van Breusegem F. (2022). Reactive oxygen species signalling in plant stress responses. Nat. Rev. Mol. Cell Biol. 23, 663–679. doi: 10.1038/s41580-022-00499-2 35760900

[B7] Noctor G. Reichheld J. P. Foyer C. H. (2018). “ROS-related redox regulation and signaling in plants,” in Seminars in cell & developmental biology, vol. 80. (New York United States: Academic Press), 3–12. 28733165 10.1016/j.semcdb.2017.07.013

[B8] Riaz A. Deng F. Chen G. Jiang W. Zheng Q. Riaz B.. (2022). Molecular regulation and evolution of redox homeostasis in photosynthetic machinery. Antioxidants 11, 2085. doi: 10.3390/antiox11112085 36358456 PMC9686623

[B9] Roy U. K. Bhattacharjee S. (2022). Exploring the parameters of central redox hub for screening salinity tolerant rice landraces of coastal Bangladesh. Sci. Rep. 12, 12989. doi: 10.1038/s41598-022-17078-2 35906294 PMC9338030

